# Circ_0001052 promotes cardiac hypertrophy via elevating Hipk3

**DOI:** 10.18632/aging.204521

**Published:** 2023-02-14

**Authors:** Mengyue Yang, Weichen Wang, Longlong Wang, Yuze Li

**Affiliations:** 1 Department of Cardiology, The First Hospital of China Medical University, Shenyang, Liaoning 110001, China

**Keywords:** circRNA, cardiac hypertrophy, Hipk3, Srsf1

## Abstract

Cardiac hypertrophy (CH) is a crucial risk factor for sudden death. Circular RNAs (circRNAs) exert significant effects in various biological and pathological processes. Circ_0001052 is sourced from Hipk3 (homeodomain-interacting protein kinase 3) and is reported to aggravate myocardial fibrosis. The purpose of the current study was to clarify the role and mechanism of circ-Hipk3 in CH. Transverse aortic constriction (TAC) was used to create an *in vivo* CH model, and angiotensin II (Ang II) therapy was used to create an *in vitro* CH model in cardiomyocytes (CMs). It was uncovered that circ_0001052 exerted pro-hypertrophic effects in Ang II-treated CMs. Next, the circular characteristics of circ_0001052 were verified, and we identified that circ_0001052 positively regulated Hipk3. Hipk3 exerted the same functions as circ_0001052 did. It is significant to note that circ_0001052 acted as the ceRNA of Hipk3 by sponging miR-148a-3p and miR-124-3p. According to rescue assays, miR-148a-3p and miR-124-3p partially reversed the effects of circ_0001052. Further, we testified that circ_0001052 recruited Srsf1 to stabilize Hipk3. Finally, rescue assays demonstrated that circ_0001052 promoted CH via up-regulation of Hipk3. In conclusion, our work unveiled that circ_0001052 promoted hypertrophic effects through elevating Hipk3 via sponging miR-148a-3p and miR-124-3p and recruiting Srsf1.

## INTRODUCTION

Pathological cardiac hypertrophy (CH) is triggered by continuous hypertrophic stresses like hypertension, ischemia and myocarditis [1]. Initially, CH is not harmful since it maintains normal cardiac function by adaptively responding to the increased cardiac load. However, extended hypertrophy motivates adjustments in metabolism, loss of adrenergic responsivity and deposition of extracellular collagen [2], which result in irreversible cardiac remodeling, eventually leading to heart failure or even sudden death [3, 4]. The *in vivo* CH model is usually established by conducting transverse aortic constriction (TAC) on mice [5]. The *in vitro* CH model has been widely reported to be established under the inducement of angiotensin II (Ang-II) [6, 7].

As a class of non-coding RNAs, circular RNAs (circRNAs) are specifically expressed in tissues [8]. CircRNAs are differentially generated by back splicing and are characterized by covalently closed continuous loops [9]. Compared with linear RNAs, circRNAs possess no free 3′ or 5′ end and have stronger stability [10]. CircRNAs regulate the expression of target genes via sponging microRNAs (miRNAs) [8] and therefore exert significant roles in various biological and pathological processes in diseases, including CH. CircSlc8a1 is a potential therapeutic target for CH via endogenously sponging miR-133a [11]. Circ-HIPK3 interacts with miR-17-3p to elevate ADCY6 expression, thereby strengthening adrenaline-mediated effects in heart failure [12].

Recently, the competitive endogenous RNA (ceRNA) pattern has attracted much attention. CircRNAs sponge miRNAs, reducing the suppression of miRNAs on target messenger RNAs (mRNAs), which is called the ceRNA mechanism. CircRNA_000203 increases the expression of Gata4 via sequestering miR-26b-5p and miR-140-3p to promote CH [7]. CircRNA HRCR enhances ARC expression by sponging miR-223, resulting in mitigated CH [13]. Circ_0076631 sponges miR-214-3p to up-regulate caspase-1, therefore attenuating cell pyroptosis in diabetic cardiomyopathy [14]. CircRNA ncx1 promotes ischemic myocardial injury through sponging miR-133a-3p to elevate CDIP1 expression [15]. CircRNA ACR regulates the Pink1/FAM65B axis to alleviate myocardial ischemia/reperfusion injury [16].

CircRNAs derived from HIPK3 have been discovered to play an important role in cardiovascular diseases. For instance, in Ang II-induced cardiac fibrosis, circRNA HIPK3 regulates the proliferation and migration of cardiac fibroblasts via sponging miR-29b-3p [17]. However, whether such circRNAs function in CH remains unclear. Here, we sought to examine the function and underlying mechanism of certain circRNA from Hipk3 in Ang II-induced CH.

## RESULTS

### Circ_0001052 promotes hypertrophic effects

**Figure 1:**
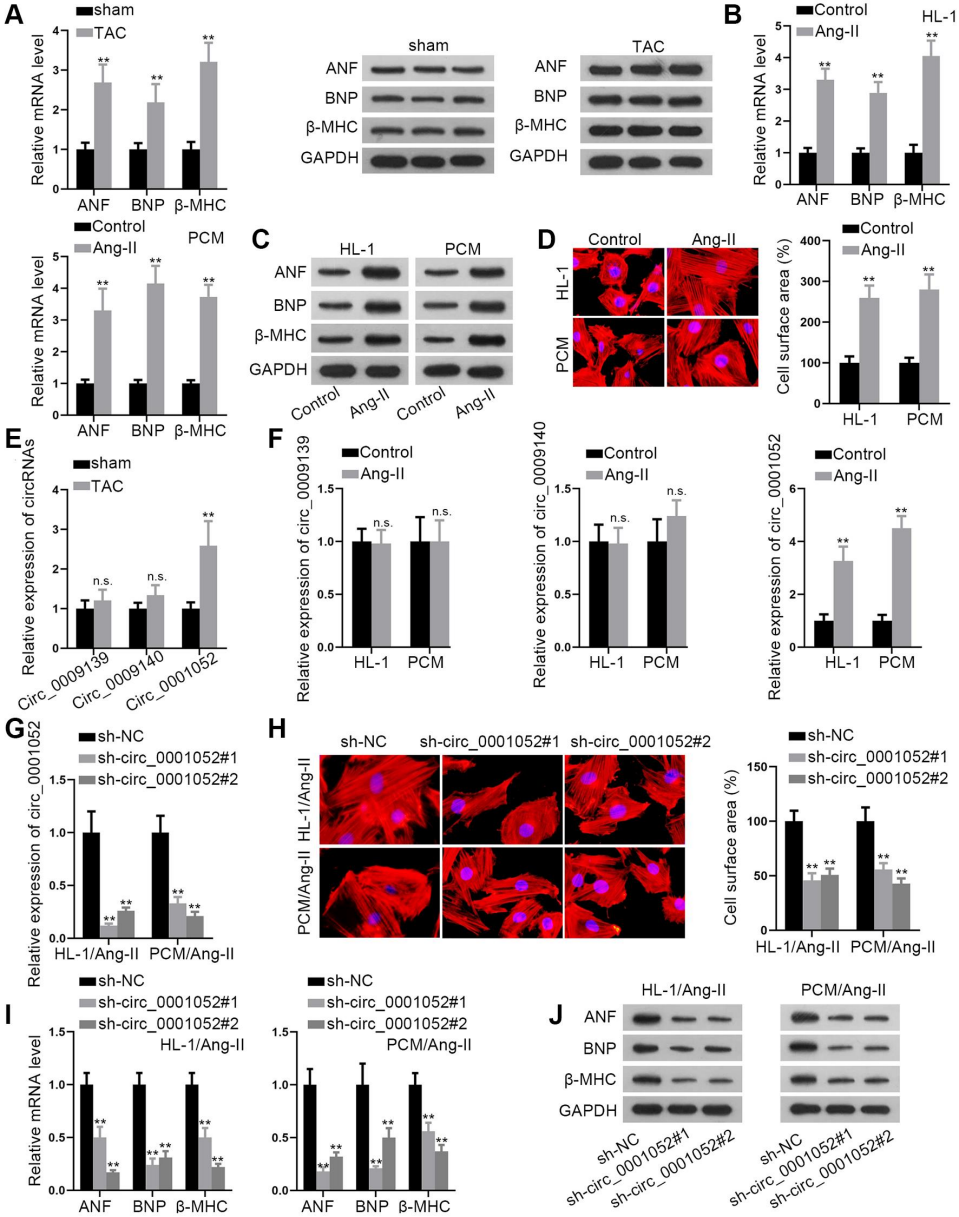
**Circ_0001052 promoted hypertrophic effects.** (**A**) The mRNA and protein levels of hypertrophic biomarkers in TAC and sham group. (**B** and **C**) The mRNA and protein levels of hypertrophic biomarkers in CMs. (**D**) The surface area of CMs by using IF staining. (**E** and **F**) The levels of circ_0009139, circ_0009140 and circ_0001052 in CMs. (**G**) The depletion efficiency of circ_0001052 in Ang II-treated CMs. (**H**) IF assay detected cell surface area under the knockdown of circ_0001052. (**I** and **J**). The mRNA and protein levels of hypertrophic biomarkers in Ang II-treated CMs with inhibited circ_0001052. ^**^P < 0.01. Abbreviation: n.s: no significance.

The first step was to establish the *in vivo* and *in vitro* CH models. As predicted, in the TAC group, ANF, BNP and β-MHC were up-regulated than sham group (Figure 1A). Accordingly, Ang II treatment significantly increased the expression of ANF, BNP, and β-MHC in HL-1 and PCM cells (Figure 1B and 1C). The cell surface area was then measured using immunofluorescence (IF). The cell surface area was enlarged in CMs treated with Ang II (Figure 1D). Since a circRNA from Hipk3 has been reported to aggravate myocardial fibrosis, we wondered if such circRNAs exerted pro-hypertrophic effects. Based on circBase [18], 3 circRNAs (mmu_circ_0009139, mmu_circ_0009140 and mmu_circ_0001052) from Hipk3 are identified. Interestingly, it was found that only mmu_circ_0001052 (named circ_0001052 for convenience in the following part of the study) was significantly up-regulated in mice after TAC (Figure 1E), as was the case in Ang II-induced CMs (Figure 1F). We then eliminated circ_0001052 in CMs treated with Ang II (Figure 1G). The conclusion showed that the depletion of circ_0001052 greatly inhibited the Ang II-induced expansion of cell surface area (Figure 1H). Similar to this, silencing circ_0001052 reduced the mRNA and protein levels of each of the three hypertrophic indicators in CMs treated with Ang II (Figure 1I and 1J). As a result, we deduced that circ_0001052 promoted the hypertrophic effects in CMs treated with Ang II.

### Circ_0001052 positively regulates Hipk3 and exerts pro-hypertrophic effects

**Figure 2:**
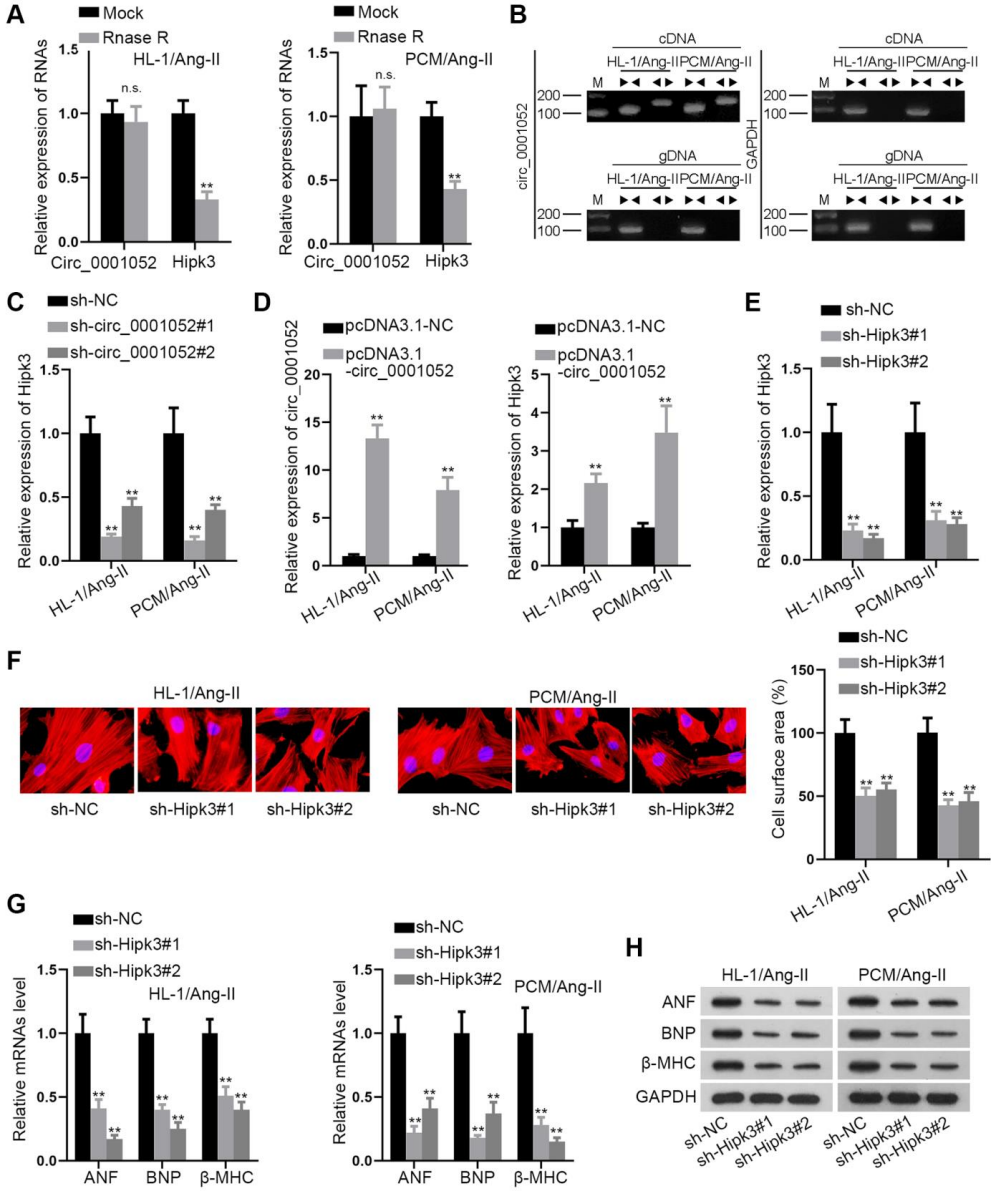
**Hipk3 was positively regulated by circ_0001052 and exerted pro-hypertrophic functions.** (**A**) The levels of circ_0001052 and Hipk3. (**B**) The circular characteristics of circ_0001052 verified by Agarose gel electrophoresis (AGE). (**C**) The mRNA levels of Hipk3 when circ_0001052 was inhibited. (**D**) The overexpression efficiency of circ_0001052 and the influence of up-regulated circ_0001052 on Hipk3 expression. (**E**) Depletion efficiency of Hipk3. (**F**) Cell surface area under Hipk3 depletion was detected by IF staining assay. (**G** and **H**) Influence of silenced Hipk3 on the expression of hypertrophic markers. ^**^P < 0.01. Abbreviation: n.s: no significance.

Then, the circular characteristics of circ_0001052 were verified. In this regard, Rnase R was used to treat cells for exploring the stability of circ_0001052 and linear Hipk3. The results revealed that Hipk3 level was obviously reduced by Rnase R treatment while circ_0001052 was hardly impacted (Figure 2A). Then divergent primers were designed for amplifying circ_0001052 while convergent primers were designed for amplifying Hipk3. The results indicated that circ_0001052 was amplified solely by divergent primers in cDNA, whereas Hipk3 was amplified by convergent primers in both cDNA and gDNA (Figure 2B). After we have verified the circular features of circ_0001052, we went on to explore whether circ_0001052 regulated the expression of its homologous gene Hipk3. As was depicted, knockdown of circ_0001052 significantly reduced the expression of Hipk3 (Figure 2C). On the contrary, enhanced expression of circ_0001052 significantly augmented Hipk3 levels in Ang II-treated CMs (Figure 2D). Furthermore, we detected the function of Hipk3 in *in vitro* CH models. The depletion efficiency of Hipk3 was firstly verified through qRT-PCR (Figure 2E). Then, the results of the IF staining assay revealed that Hipk3 depletion significantly reduced cell surface area (Figure 2F). Also, the levels of ANF, BNP and β-MHC were remarkably decreased in response to the depletion of Hipk3 (Figure 2G and 2H). In brief, Hipk3 was positively modulated by circ_0001052 and promoted hypertrophic effects in CMs.

### Circ_0001052 competes with Hipk3 to bind to miR-148a-3p and miR-124-3p

**Figure 3:**
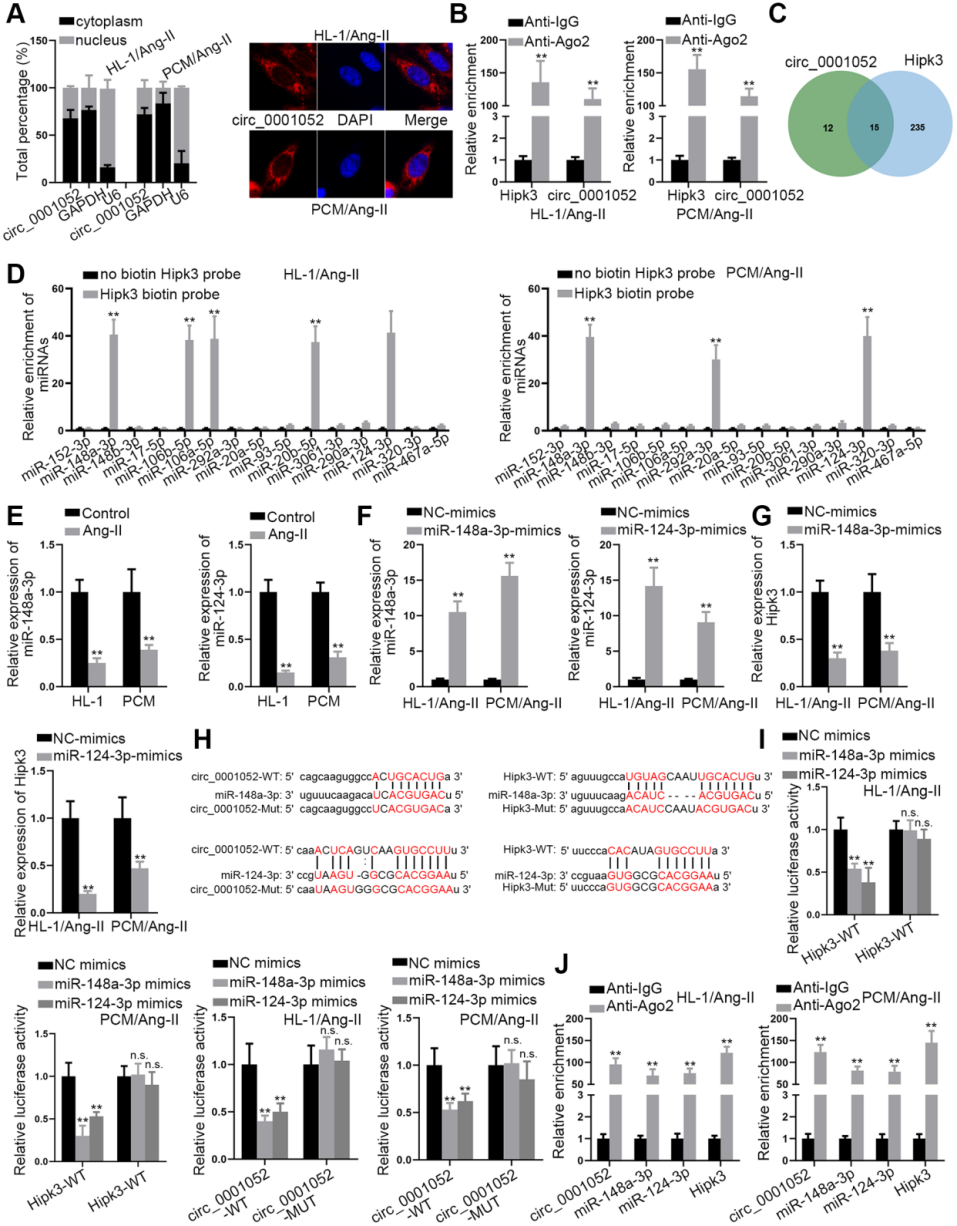
**Circ_0001052 competed with Hipk3 to interact with miR-148a-3p and miR-124-3p.** (**A**) The subcellular location of circ_0001052. (**B**) RIP assay revealed the relative enrichment of circ_0001052 and Hipk3 in Ago2 or IgG group. (**C**) StarBase database predicted 15 miRNAs binding to both circ_0001052 and Hipk3. (**D**) RNA Pull down assay illustrated the relative enrichment of 15 candidate miRNAs pulled down by biotin labeled Hipk3. (**E**) The levels of miR-148a-3p/miR-124-3p in Ang II-treated CMs. (**F**) The overexpression efficiency of miR-148a-3p and miR-124-3p. (**G**) Influence of up-regulated miR-148a-3p or miR-124-3p on Hipk3 expression. (**H**) Binding sites between circ_0001052/Hipk3 and miR-148a-3p/miR-124-3p. (**I**) The luciferase activity of circ_0001052-WT/Mut or Hipk3-WT/Mut in response to the up-regulation of miR-148a-3p or miR-124-3p. (**J**) RIP assay tested the enrichment of circ_0001052, miR-148a-3p, miR-124-3p and Hipk3 in Ago2 or IgG group. ^**^P < 0.01. Abbreviation: n.s: no significance.

In this section, how circ_0001052 regulated Hipk3 was explored. To investigate the cellular location of circ_0001052 in Ang II-treated CMs, subcellular fraction and FISH experiments were carried out. Circ_0001052 was distributed in both nuclear and cytoplasmic fractions, with a greater percentage in the latter (Figure 3A). Next, the results of the Ago2-RIP test showed that Hipk3 and circ_0001052 were considerably enriched in the Ago2-assembled RNA-induced silencing complex (RISC) (Figure 3B), suggesting that they could be involved in a ceRNA network. In order to find common miRNAs that combine with both circ_0001052 and Hipk3, starBase database was searched [19]. As was illustrated in the Venn diagram, 15 miRNAs were revealed (Figure 3C). We conducted the RNA pull down assay using biotin-labeled Hipk3 to pull down these 15 miRNAs. It manifested that 5 miRNAs were pulled down in HL-1/Ang-II and 3 miRNAs were pulled down in PCM/Ang-II, with 2 miRNAs (miR-148a-3p and miR-124-3p) shared in both cells (Figure 3D). MiR-148a-3p and miR-124-3p levels were shown to have dramatically decreased in Ang II-induced CMs (Figure 3E). In addition, using miR-148a-3p/miR-124-3p mimics, we increased the expression of these two miRNAs (Figure 3F). As expected, increased levels of miR-148a-3p or miR-124-3p greatly hindered Hipk3 expression (Figure 3G). Moreover, the binding sequences of miR-148a-3p/miR-124-3p and circ_0001052/Hipk3 were obtained from starBase database, with the sequence of circ_0001052-Mut/Hipk3-Mut contained mutant binding sites showed as well (Figure 3H). It was discovered that the up-regulation of miR-148a-3p or miR-124-3p reduced the luciferase activity of circ_0001052-WT or Hipk3-WT but had no effect on the luciferase activity of circ_0001052-Mut or Hipk3-Mut (Figure 3I). Finally, it was certified that circ_0001052, miR-148a-3p, miR-124-3p and Hipk3 were all significantly enriched in RISC (Figure 3J). Therefore, through sponging miR-148a-3p and miR-124-3p, circ_0001052 acted as the ceRNA of Hipk3.

### Co-inhibition of miR-148a-3p and miR-124-3p partially rescues the effects of silenced circ_0001052 on Ang II-induced CMs

**Figure 4:**
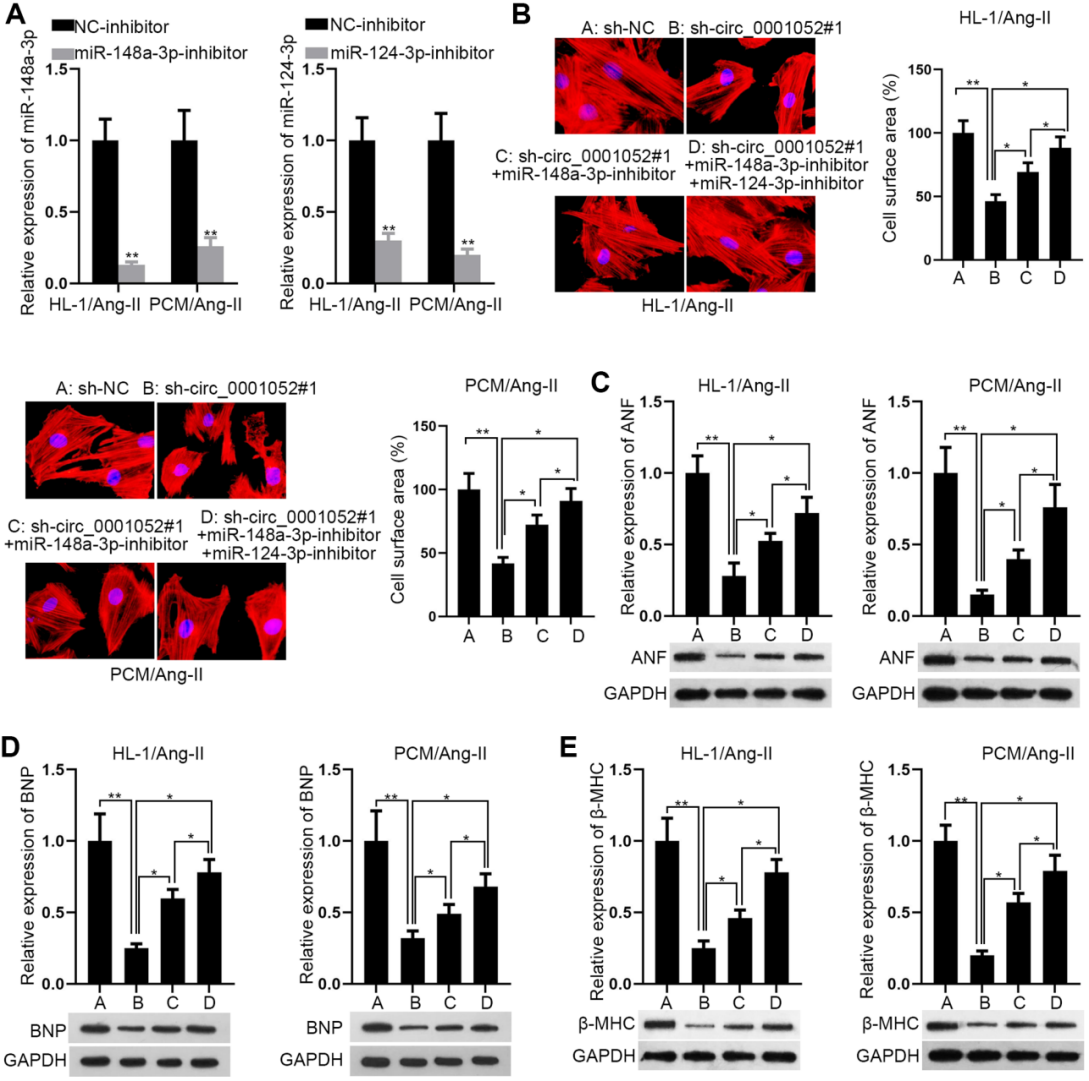
**MiR-148a-3p/miR-124-3p partially mediated the effects of circ_0001052 in CMs.** (**A**) Depletion efficiency of miR-148a-3p and miR-124-3p. (**B**) The rescue effects of miR-148a-3p inhibition or together with miR-124-3p inhibition on the surface area of circ_0001052-silenced CMs. (**C**–**E**) The levels of hypertrophic biomarkers in indicated CMs. ^*^P < 0.05, ^**^P < 0.01.

The rescue experiments were carried out to determine whether circ_0001052 affected CH via miR-148a-3p and miR-124-3p. Prior to that, the depletion efficacies of miR-148a-3p and miR-124-3p were validated for the following assays (Figure 4A). Intriguingly, both miR-148a-3p and miR-124-3p inhibition partially reversed the suppressive effects of silenced circ_0001052 on cell surface area (Figure 4B). Similarly, circ_0001052 deficiency-induced downregulation of hypertrophic markers was partly recovered by co-suppression of miR-148a-3p and miR-124-3p (Figure 4C–4E). We concluded that miR-148a-3p/miR-124-3p partially mediated the effects of circ_0001052, which indicated that circ_0001052 regulated Hipk3 in another way.

### Circ_0001052 recruites Srsf1 to stabilize Hipk3

**Figure 5:**
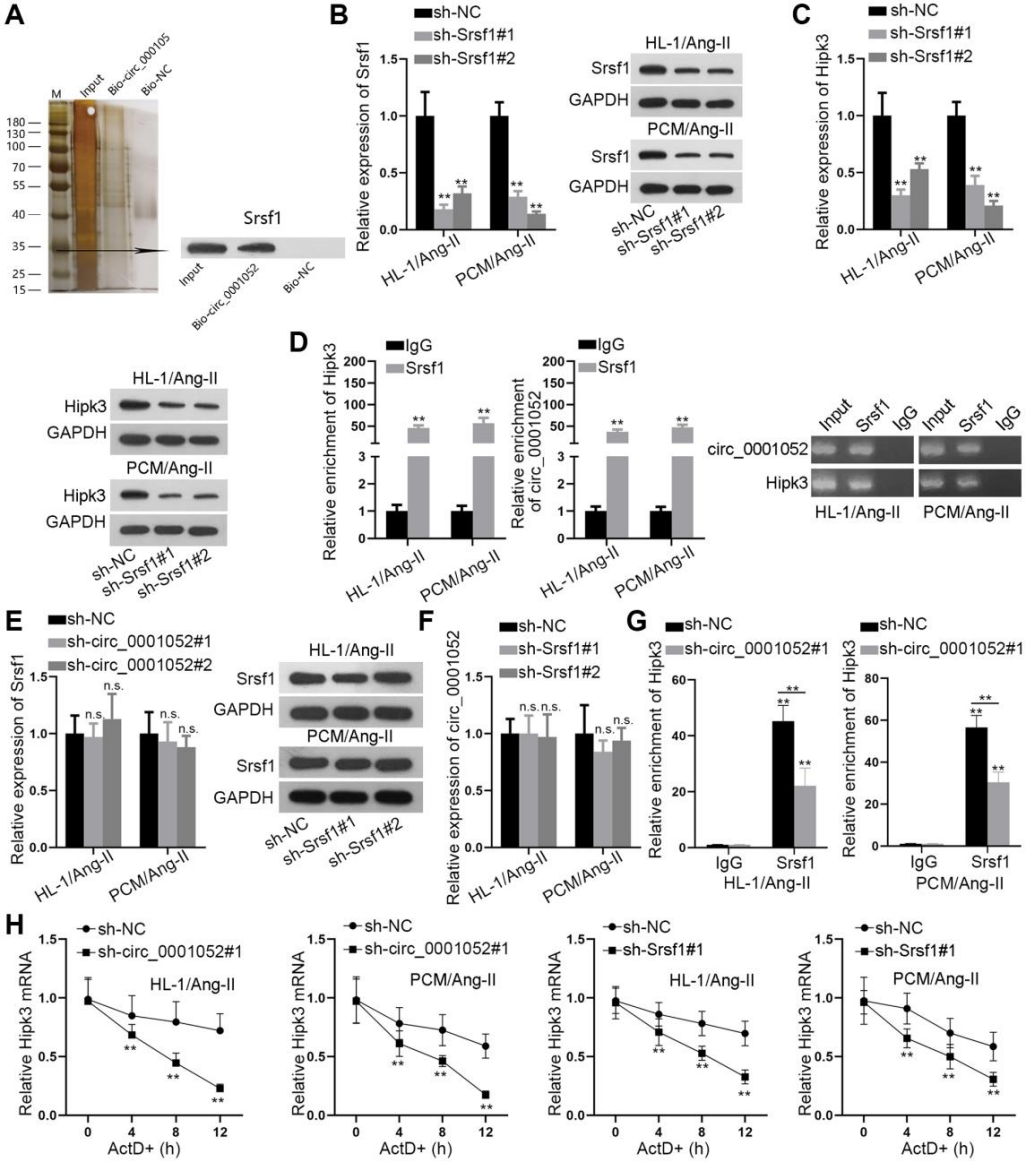
**Circ_0001052 recruited Srsf1 to stabilize Hipk3.** (**A**) RNA pull down assay, mass spectrometry and western blot analyses revealed Srsf1 as the RBP for circ_0001052. (**B**) Depletion efficiency of Srsf1 in Ang-II induced CMs. (**C**) The expression of Hipk3 with Srsf1 depletion. (**D**) RIP assay followed by AGE revealed the enrichment of Hipk3 and circ_0001052 in anti-IgG or anti-Srsf1 group. (**E**) Influence of silenced circ_0001052 on Srsf1 expression. (**F**) The impact of silenced Srsf1 on circ_0001052 expression. (**G**) RIP assay revealed the enrichment of Hipk3 pulled down by anti-Srsf1 when circ_0001052 was silenced. (**H**) The mRNA level of Hipk3 under ActD treatment when circ_0001052 or Srsf1 was silenced. ^**^P < 0.01. Abbreviation: n.s: no significance.

CircRNAs indirectly regulate the level of target genes by binding to RNA-binding proteins (RBPs). Hence, we wondered if circ_0001052 recruited certain RBP to stabilize Hipk3. Firstly, we conducted RNA pull down assay to identify the potential RBP that bound to circ_0001052. According to the results of mass spectrometry, Srsf1 was significantly pulled down by Bio-circ_0001052 (Figure 5A). Also, Srsf1 was predicted to have the potential to bind with circ_0001052 based on starBase database. Next, we silenced Srsf1 in both Ang II-treated HL-1 and PCM cells (Figure 5B), and discovered that depletion of Srsf1 significantly reduced the expression of Hipk3 (Figure 5C). Next, the outcomes of RIP assays disclosed that both Hipk3 and circ_0001052 were obviously pulled down by anti-Srsf1 (Figure 5D). Then, we revealed that Srsf1 expression was not impacted by circ_0001052 depletion (Figure 5E). Also, silencing Srsf1 had no influence on circ_0001052 expression (Figure 5F). Further, it was revealed that when circ_0001052 was silenced, the enrichment of Hipk3 in anti-Srsf1 groups was significantly reduced in Ang II-treated CMs (Figure 5G). Of note, we validated that when circ_0001052 or Srsf1 was silenced, the resistance of Hipk3 to ActD treatment was significantly mitigated (Figure 5H). In summary, circ_0001052 served as the scaffold to recruit Srsf1, therefore stabilizing Hipk3 mRNA in CMs.

### Circ_0001052 serves pro-hypertrophic effects via up-regulation of Hipk3

**Figure 6:**
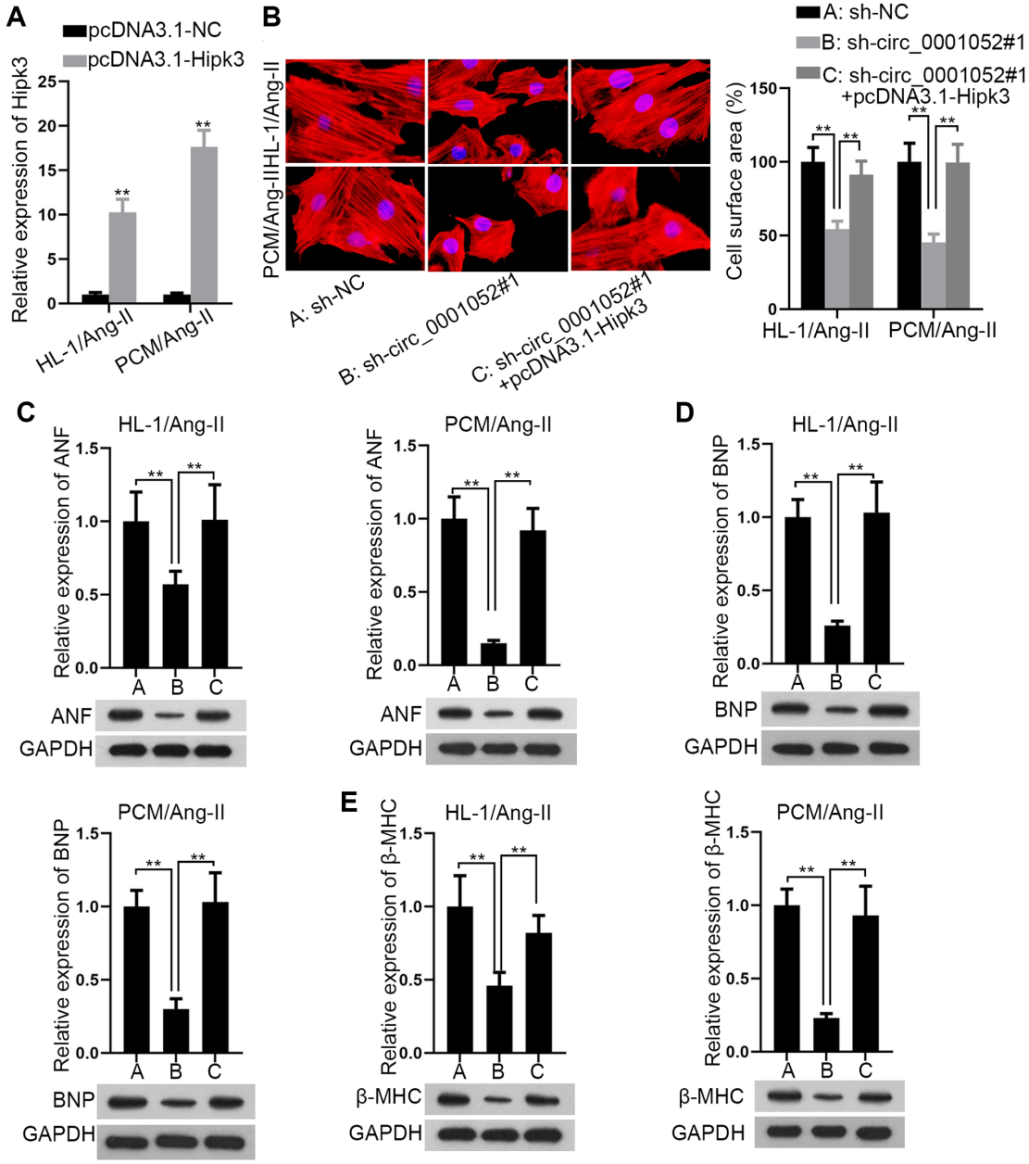
**Circ_0001052 played a pro-hypertrophic part in CMs via targeting Hipk3.** (**A**) Overexpression efficiency of Hipk3. (**B**) The cell surface area in circ_0001052-silenced CMs with up-regulated Hipk3. (**C**–**E**). The levels of hypertrophic biomarkers in CMs under diverse contexts. ^**^P < 0.01.

Finally, rescue assays were carried out to explore whether Hipk3 was required in circ_0001052-regulated CH. Expression level of Hipk3 was firstly verified (Figure 6A). Up-regulation of Hipk3 absolutely rescued circ_0001052 inhibition-mediated suppressive effects on cell surface area (Figure 6B). In the meantime, the repressive influence of silenced circ_0001052 on the expression of three hypertrophic markers was completely counteracted by up-regulated Hipk3 (Figure 6C–6E). All in all, elevated expression of Hipk3 completely rescued the anti-hypertrophic effects of silenced circ_0001052 on Ang II induced CMs.

**Figure 7:**
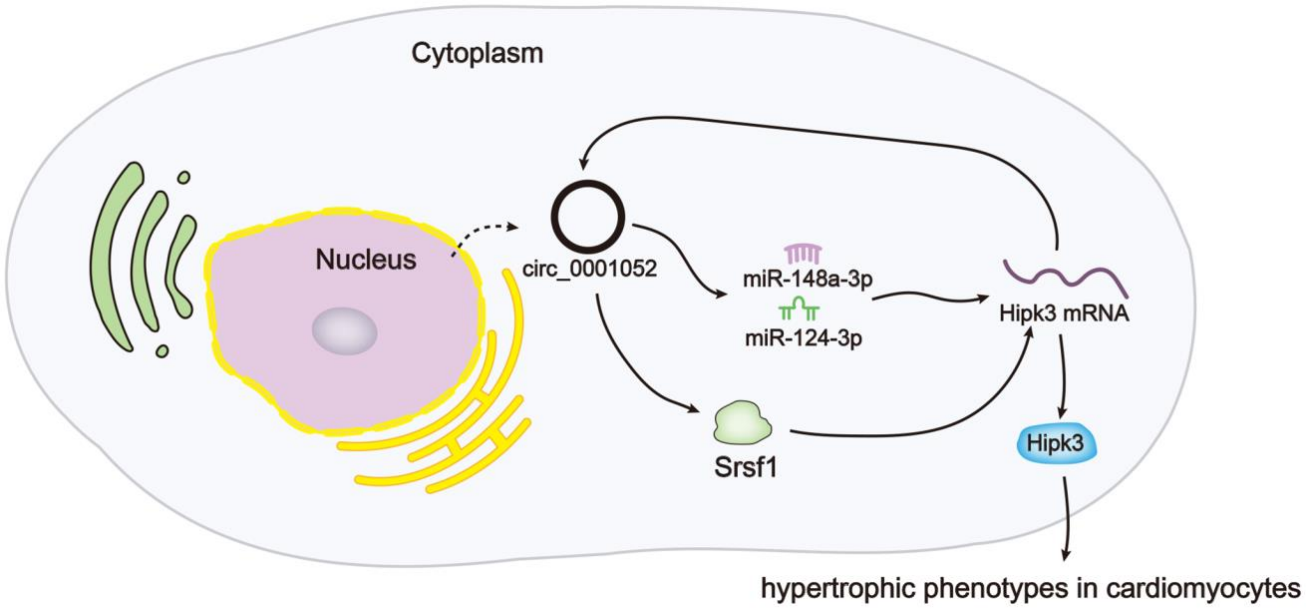
Schematic abstract revealed the mechanism underlying circ_0001052 regulated Hipk3 in CMs.

Based on all the findings in this work, we drew a conclusion that circ_0001052, a circRNA derived from Hipk3, boosted Hipk3 expression via sequestering miR-148a-3p/miR-124-3p and recruiting Srsf1, therefore facilitating hypertrophic phenotypes in CMs (Figure 7).

## DISCUSSION

CircRNAs generated from human HIPK3 (circ-HIPK3) have been shown to play important roles in cardiovascular disease. Circ-HIPK3, for example, exacerbates the effect of adrenaline on heart failure through modulating the miR-17-3p/ADCY6 axis [12]. Circ-HIPK3 contributes to the differentiation of myoblasts [20]. High glucose induces circ-HIPK3 downregulation in human umbilical vein endothelial cells [21]. Also, circRNA from Hipk3 (circ-Hipk3) was suggested to aggravate myocardial fibrosis via sponging miR-29b-3p [17]. Present study verified that a novel circRNA from Hipk3, circ_0001052, was considerably up-regulated in both *in vivo* and *in vitro* CH models. Silencing circ_0001052 impaired hypertrophic effects in Ang II-induced CMs.

Mounting evidence has indicated that circRNAs have the potential to regulate their homologous genes. For instance, circRNA-ENO1 elevates ENO1 expression to enhance glycolysis and tumor progression in lung adenocarcinoma [22]. CircABCC2 modulates ABCC2 expression via endogenously sponging miR-665 [23]. CircRUNX2 elevates RUNX2 expression and attenuates osteoporosis by sponging miR-203 [24]. In the present study, we consistently revealed that circ_0001052 positively regulated its host gene Hipk3. Also, Hipk3 exerted pro-hypertrophic effects in Ang II-induced CMs.

Previous studies have largely proposed the ceRNA pattern as a potential way for circRNAs to modulate their host genes [23]. In this work, we uncovered that both circ_0001052 and Hipk3 were abundantly enriched in the RISC. Further, miR-148a-3p and miR-124-3p were validated as the shared miRNAs by both circ_0001052 and Hipk3. Former reports indicated that miR-148a-3p suppresses IKBKB to inactivate NF-κB signaling in human aortic valve cells [25]. MiR-148b-3p is directly associated with Epicardial adipose tissue [26]. MiR-124-3p is a survival-predictor for patients with cardiac arrest out of hospital [27]. LncRNA ROR modulates ischaemia reperfusion injury-induced inflammatory response in human CMs via sponging miR-124-3p and up-regulating TRAF6 [28]. Besides, the ceRNA networks involving circRNAs and miR-148a-3p or miR-124-3p have also been reported in several previous studies. CircANKS1B serves as the ceRNA of USF1 by sponging miR-148a-3p and miR-152-3p [29]. CircRNA_005186 is a sponge for miR-124-3p and regulates the expression of Epha2 [30]. The present study indicated that miR-148a-3p and miR-124-3p were involved in the regulation of circ_0001052 on Hipk3. Intriguingly, the co-inhibition of miR-148a-3p and miR-124-3p partially rescued the effects of silenced circ_0001052 on the hypertrophic phenotypes of Ang II-induced CMs.

As supported by numerous researches, circRNAs can also interact with RBP to promote or inhibit the expression of protein-coding genes. For instance, circ-PABPN1 binds to HuR protein and prevents the binding of HuR to PABPN1 mRNA, thus reducing PABPN1 translation [31]. CircRNA ZKSCAN1 interacts with FMRP to prevent the binding of FMRP with CCAR1 complex in hepatocellular carcinoma [32]. Circ-HuR interacts with CNBP protein to restrain its binding to HuR promoter in gastric cancer cells [33]. In present study, we uncovered that circ_0001052 served as the scaffold of Srsf1 to stabilize Hipk3. Srsf1 was reported to increase the stability of mRNAs. CircRNA SMARCA5 binds to SRSF1 protein to modulate VEGFA mRNA splicing in glioblastoma multiforme [34]. Also, SRSF1 could protect DBF4B from DNA damage via modulation on pre-mRNA splicing [35]. Current study revealed that circ_0001052 recruited Srsf1 protein to stabilize Hipk3 mRNA and therefore elevate Hipk3 expression. Of note, we proved that Hipk3 overexpression completely offset the impact of silenced circ_0001052 on Ang II-induced CMs.

Although we have fully demonstrated that circ_0001052 promoted cardiac hypertrophy, there are still some limitations in our study. Firstly, the evidence from animal experiments is insufficient, and there is a lack of histological and morphological evidence. Secondly, we have only confirmed that circ_0001052 promoted cardiac hypertrophy through the ceRNA mechanism. The deeper mechanism remains unknown, such as the effect of circ_0001052 on cell signaling pathways and the cell metabolic cycle.

In conclusion, present study uncovered that circ_0001052 sponged miR-148a-3p/miR-124-3p and recruited Srsf1 protein to boost Hipk3 level, finally aggravating hypertrophic phenotypes in CMs. Of importance, these findings indicated circ_0001052 as a putative biomarker for CH treatment.

## METHODS

### Animal study

This animal study protocol was approved and supervised by the Animal Ethics Committee of the First Hospital of China Medical University. C57BL6 mice (male; 8 weeks) were used to create an *in vivo* CH model via transverse aortic constriction (TAC). Mice in the sham group were experienced the same as the TAC group, except aorta seam.

### Cell culture and treatments

The isolated primary CMs (PCM) and mouse CMs HL-1 (ATCC; Manassas, VA, USA) were cultured in DMEM (Gibco, Grand Island, NY, USA) with 10% FBS (Gibco) and 1% penicillin/streptomycin (Gibco) at 37°C with 5% CO_2_. 1 mmol/L of angiotensin II (Ang-II; Sigma-Aldrich) was used to establish an *in vitro* CH model.

### Quantitative real-time PCR (qRT-PCR)

Cells were treated with Trizol reagent (Invitrogen, Carlsbad, CA, USA) to extract total RNA, then total RNA was reverse-transcribed into cDNA by the Reverse Transcription Kit (Invitrogen). SYBR-Green Real-Time PCR Systems (Invitrogen) were used to progress qRT-PCR. Using GAPDH/U6 as the endogenous control, relative RNA expression was evaluated.

### Western blot

Cells were treated with RIPA lysis buffer (Beyotime, Shanghai) to extract the total protein, which was then separated by 12% SDS-PAGE (Bio-Rad, Hercules, CA, USA) and transferred to PVDF membranes (Millipore, Bedford, MA, USA). Membranes were then interacted with the matching primary antibodies (Abcam, Cambridge, MA, USA) for ANP (ab225844), BNP (ab236101), β-MHC (ab172967), Srsf1 (ab129108), Hipk3 (ab72538), and the loading control GAPDH(ab181602) for the duration of the night at 4°C. Later, for a total of two hours, secondary antibodies (ab205718, Abcam) were added. All the primary antibodies were diluted 1000 times, and the secondary antibodies 10,000 times.

### Immunofluorescence (IF) staining

CMs were washed twice using phosphate-buffered saline (PBS; Sigma-Aldrich), followed by fixing in 4% paraformaldehyde (PFA; Sigma-Aldrich) for 20 min and washing thrice in PBS. Then CMs were blocked with 1% bovine serum albumin (BSA; Sigma-Aldrich). Sequentially, cells were treated with anti-actin (ab7817; Abcam) and secondary antibody (ab150117, Abcam). It was decided to use DAPI (Sigma-Aldrich) for nucleus labeling. A fluorescent microscope was used to measure the size of the cell surface (Zeiss, Jena, Germany).

### Plasmid transfection

The particular shRNAs for the genes Hipk3, Srsf1, and circ_0001052, as well as their corresponding control shRNAs, were purchased from GenePharma (Shanghai, China). Besides, we got the pcDNA3.1 vector targeting Hipk3 and the pcDNA3.1 (+) CircRNA Mini Vector targeting circ_0001052 from GenePharma. Genechem created the miR-148a-3p mimics/inhibitors and the miR-124-3p mimics/inhibitors (Shanghai, China). These plasmids were appropriately transfected into CMs using Lipofectamine 2000 (Invitrogen).

### Subcellular fraction assay

The nuclear and cytoplasmic fractions of CMs were obtained by using Nuclear/cytoplasmic fractionation PARIS Kit (Thermo Fisher Scientific, Waltham, MA, USA). Then qRT-PCR was carried out to assess the concentration of circ_0001052 in various fractions, using GAPDH and U6 as fractionation markers.

### FISH assay

The specific probe of circ_0001052 was designed by Ribobio (Guangzhou, China) and used as per the direction. The fixed CMs were dehydrated and cultivated with FISH-probe in hybridization buffer. Following DAPI staining, samples were examined under a fluorescence microscope (Zeiss). The sequence of circ_0001052 probe was as follow: 5′-GAGGCCAUACCUGUAGUAGCG-3′.

### RNA immunoprecipitation (RIP)

RIP assay was carried out in CMs according to the instructions of the Magna RIP RNA-Binding Protein Immunoprecipitation Kit (Millipore). Anti-Ago2 antibody (03-110, Sigma-Aldrich) or a control anti-IgG antibody (ab190475, Abcam) were used for immunoprecipitation, which was then completed with the addition of magnetic beads. The precipitated RNAs were ultimately examined using qRT-PCR.

### RNA pull down assay

Hipk3 or circ_0001052 biotinylated RNA probes were incubated with the lysates of CMs, and then magnetic beads were added and incubated for an additional hour. The RNAs or proteins in pull-downs were analyzed using qRT-PCR or western blotting.

### Luciferase reporter assay

Using pmirGLO dual-luciferase vectors (Promega, Madison, WI, USA), the Hipk3 3′UTR or circ_0001052 fragments encompassing the miR-148a-3p or miR-124-3p target sequences (wild-type or mutant) were used to create Hipk3-WT/MUT or circ_0001052-WT/MUT. MiR-148a-3p, miR-124-3p, or NC mimics were co-transfected into CMs with the obtained constructs. Dual luciferase reporter assay system was used to check the luciferase activity.

### Statistical analyses

The mean and standard deviation of the data were displayed. With *p* < 0.05 serving as the significant level, differences between groups were examined using the Student’s *t* test or one-way ANOVA. Experiments were implemented at least 3 times.
